# PARP1 inhibitor olaparib (Lynparza) exerts synthetic lethal effect against ligase 4-deficient melanomas

**DOI:** 10.18632/oncotarget.12270

**Published:** 2016-09-27

**Authors:** Małgorzata Czyż, Monika Toma, Anna Gajos-Michniewicz, Kinga Majchrzak, Grazyna Hoser, Janusz Szemraj, Margaret Nieborowska-Skorska, Phil Cheng, Daniel Gritsyuk, Mitchell Levesque, Reinhard Dummer, Tomasz Sliwinski, Tomasz Skorski

**Affiliations:** ^1^ Department of Molecular Biology of Cancer, Medical University of Lodz, 92-215 Lodz, Poland; ^2^ Department of Molecular Genetics, University of Lodz, 90-236 Lodz, Poland; ^3^ Department of Flow Cytometry, Medical Center for Postgraduate Education, 01-813 Warsaw, Poland; ^4^ Department of Medical Biochemistry, Medical University of Lodz, 92-215 Lodz, Poland; ^5^ Department of Microbiology and Immunology, Temple University Lewis Katz School of Medicine, Philadelphia, PA 19140, USA; ^6^ Department of Dermatology, Faculty of Medicine, University Hospital Zürich, and University of Zürich, CH-8952, Zürich, Switzerland

**Keywords:** melanoma, PARP1 inhibitor, synthetic lethality

## Abstract

Cancer including melanoma may be “addicted” to double strand break (DSB) repair and targeting this process could sensitize them to the lethal effect of DNA damage. PARP1 exerts an important impact on DSB repair as it binds to both single- and double- strand breaks. PARP1 inhibitors might be highly effective drugs triggering synthetic lethality in patients whose tumors have germline or somatic defects in DNA repair genes. We hypothesized that PARP1-dependent synthetic lethality could be induced in melanoma cells displaying downregulation of DSB repair genes. We observed that PARP1 inhibitor olaparib sensitized melanomas with reduced expression of DNA ligase 4 (LIG4) to an alkylatimg agent dacarbazine (DTIC) treatment *in vitro*, while normal melanocytes remained intact. PARP1 inhibition caused accumulation of DSBs, which was associated with apoptosis in LIG4 deficient melanoma cells. Our hypothesis that olaparib is synthetic lethal with LIG4 deficiency in melanoma cells was supported by selective anti-tumor effects of olaparib used either alone or in combination with dacarbazine (DTIC) in LIG4 deficient, but not LIG4 proficient cells. In addition, olaparib combined with DTIC inhibited the growth of LIG4 deficient human melanoma xenografts. This work for the first time demonstrates the effectiveness of a combination of PARP1 inhibitor olaparib and alkylating agent DTIC for treating LIG4 deficient melanomas. In addition, analysis of the TCGA and transcriptome microarray databases revealed numerous individual melanoma samples potentially displaying specific defects in DSB repair pathways, which may predispose them to synthetic lethality triggered by PARP1 inhibitor combined with a cytotoxic drug.

## INTRODUCTION

While melanomas can be successfully treated in the early stages, the appearance of metastasis in distant organs worsens prognosis and drops median survival below nine months [1]. Despite of the recent advances in melanoma treatment, including immunotherapies and targeted therapies, a resistance is developed in the majority of patients [2] indicating that genotoxic therapies might still be needed. It has been suggested that cancer cells survive genotoxic stress due to acquired abnormalities in DNA repair system [3]. The 'addiction' of cancer cells to compensatory DNA repair mechanisms, especially double strand break (DSB) repair, may create an opportunity to target these pathways to eliminate malignant cells [3, 4].

DSBs are highly cytotoxic DNA lesions caused by reactive oxygen species (ROS), ionizing radiation and genotoxic drugs [4]. In proliferating cells DSBs are usually repaired by two major mechanisms, BRCA1/BRCA2-dependent homologous recombination (HR) and DNA-PKcs-mediated non-homologous end-joining (D-NHEJ), whereas PARP1-dependent back-up NHEJ (B-NHEJ) serves as an alternate mechanism [5–7]. In addition, PARP1 may decrease the number of potentially lethal DSBs, either by stimulation of base excision repair (BER) and single-strand break (SSB) repair and/or by facilitation of MRE11-mediated recruitment of RAD51, as well as, by involvement in relocation of XRCC1, an essential protein for an effective DSB repair and restart of stalled replication forks [8, 9].

**Figure 1:**
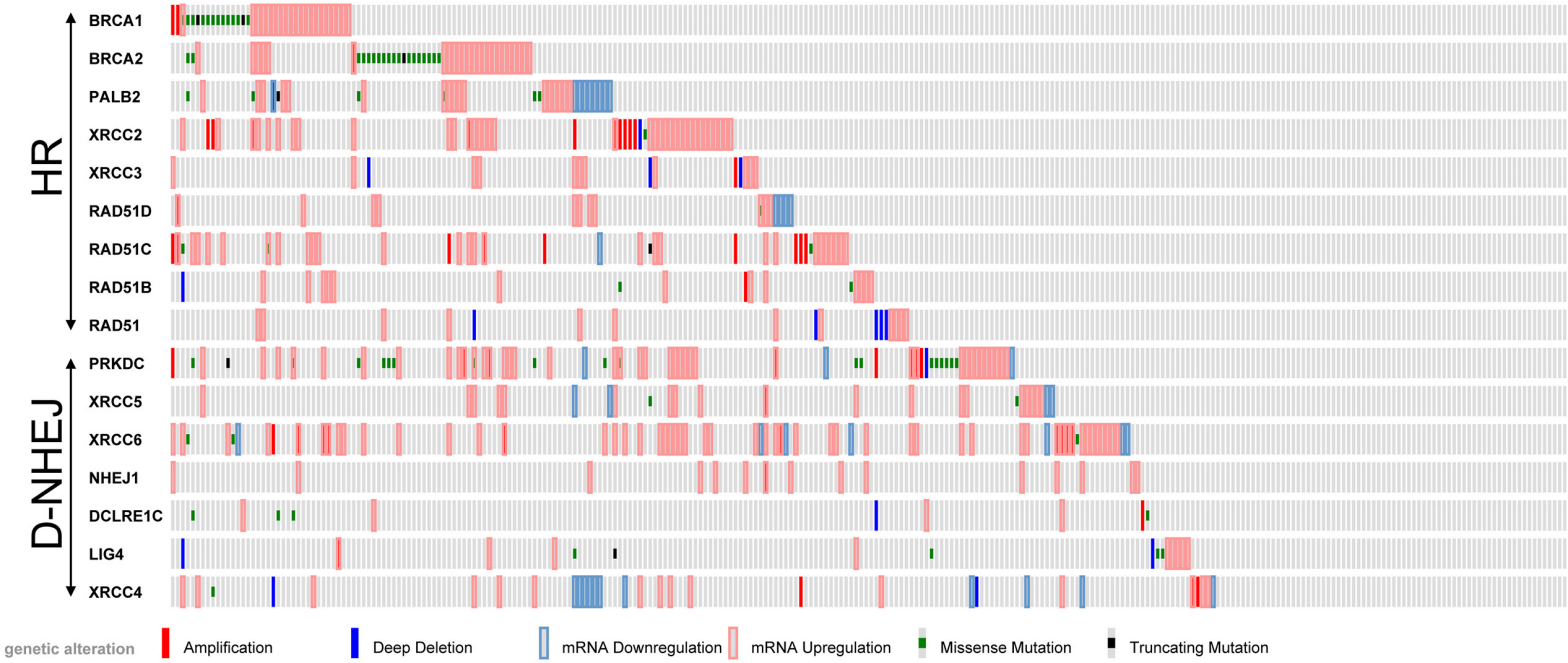
Analysis of TCGA database of 287 individual skin cutaneous melanomas Deregulated expression (Z-score >2.0) and/or mutations of the genes in DSB repair pathways, HR and D-NHEJ, are shown.

It was reported that cells deficient in BRCA1/BRCA2-mediated HR are sensitive to PARP1 inhibitors, such as the recently FDA approved olaparib (Lynparza, Astra-Zeneca) due to induction of synthetic lethality [10]. Since TCGA database analysis revealed that melanoma samples display deregulated expression and/or mutations of the genes encoding DSB repair proteins (Figure 1), we hypothesize that DSB repair deficiencies could sensitize individual melanomas to PARP1 inhibitor administered either alone or in combination with DSB-inducing genotoxic agents, such as dacarbazine (DTIC) [11].

## RESULTS

### Genes involved in the DSB repair pathway are differentially expressed in patient-derived melanoma cells and in normal melanocytes

**Figure 2:**
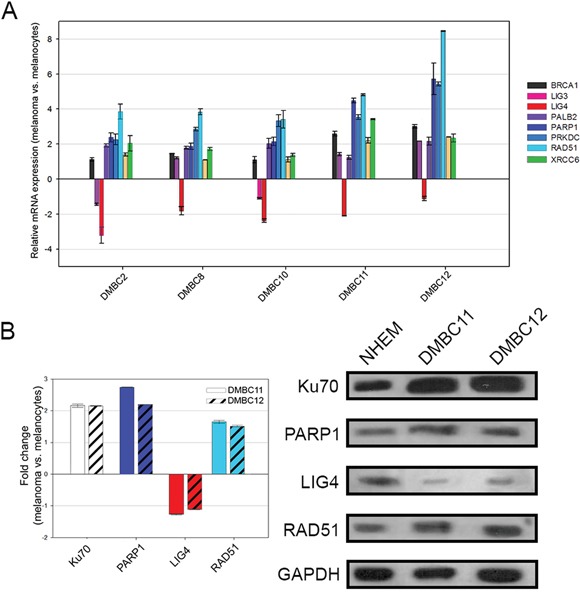
Expression profiles of DNA double-strand break repair genes in melanoma cells compared to melanocytes **A.** The transcript level of each gene was normalized to the expression of a reference gene (18S RNA). Data is presented as fold change in melanoma cells versus melanocytes, in which expression levels of the genes were set as 1. The mean values ± SD were calculated from 3 experiments performed in triplicates. **B.** The protein level was normalized to the expression of a reference protein, GAPDH. Data is presented as fold change in melanoma cells versus melanocytes, in which the expression levels of the proteins were set as 1. The means ± SD were calculated from 3 experiments. Representative Western blot results are included.

To test the potential anti-melanoma effect of PARP1 inhibitors we established six patient-derived melanoma cell lines. Real-time PCR was used to determine the gene expression profile in melanoma cells and in normal human melanocytes. Eight genes were examined, whose products are essential for DSB repair pathways (BRCA1, PALB2, and RAD51 in HR; PRKDC, XRCC6, and LIG4 in D-NHEJ; PARP1 and LIG3 in B-NHEJ). Significant differences were found in the gene expression profiles between melanoma cells and melanocytes. In particular, all melanoma lines showed a decreased level of DNA ligase 4 (*LIG4*) (Figure 2A).

Protein expression status of LIG4, RAD51, PARP1, Ku70 was determined by Western blot analysis in normal melanocytes and melanoma cell lines (DMBC11, DMBC12) (Figure 2B). Both DMBC11 and DMBC12 cell lines displayed elevated expression of RAD51, PARP1 and Ku70 proteins, whereas expression of LIG4 was downregulated.

### Olaparib used either alone or in combination with DTIC induced cytotoxic effects in patient-derived LIG4-deficient melanoma cells

**Figure 3:**
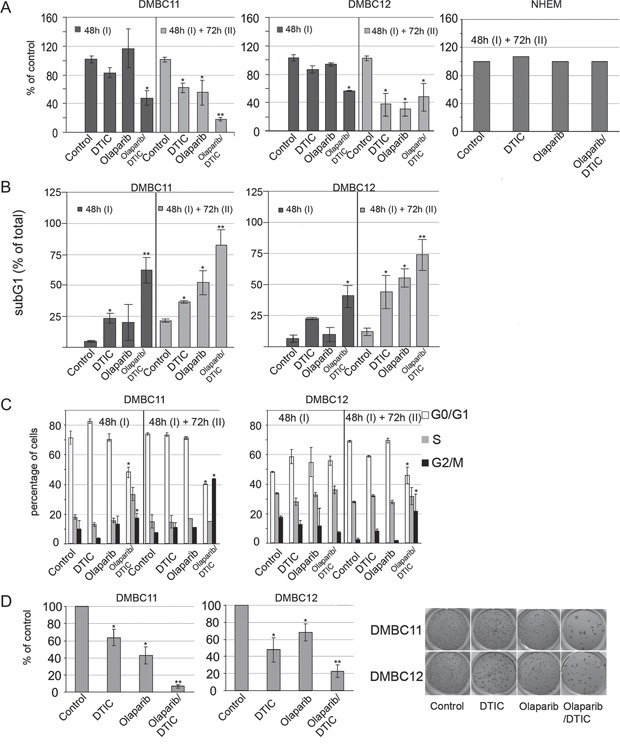
Effects of olaparib and DTIC, used alone or in combination, on viability, distribution in cell cycle and clonogenicity of melanoma cells **A.** Viability was measured using PI staining and flow cytometry, and it is shown as % of vehicle control. Means ± SD of 2 independent experiments performed in triplicates are shown. **B.** Cell death was measured by accumulation of melanoma cells in the sub-G1 fraction; mean ± SD of 2 independent experiments. **C.** Distribution of melanoma cells through the cell-cycle phases was analyzed by flow cytometry. Left panel, bars represent cell distribution after 48 hours and after additional 72 hour treatments of DMBC11 and DMBC12 populations with DTIC and olaparib, used alone or in combination. ModFit LT 3.0 software was used to calculate the percentages of cells in each fraction; means ± SD of two independent experiments are shown. Right panel, representative histograms of DMBC11 cells treated with two doses of indicated drugs (48 hours followed by 72 hours). **D.** Clonogenic assay showing the long-term effects olaparib and/or DTIC on melanoma cell lines DMBC11 and DMBC12. Left panel, bars represent clonogenic efficiency in drug-treated melanoma cell populations, expressed as percentages of clonogenic efficiency in vehicle-treated control; mean ± SD of 2 independent experiments. Right panel, photographs of a representative experiment are shown.

To determine the influence of tested compounds on viable cell number, plasma membrane integrity was measured by cytometric analysis (Figure 3A). After the first 48 hours of treatment, only the combination of olaparib and DTIC markedly reduced viability reaching about 54% of control. The second dose and additional incubation for 72 hours induced cell response to drugs, used either alone or in combination. Normal melanocytes were not affected by the treatments.

Cell death was assessed by the appearance of sub-diploid fraction (subG1, Figure 3B). Sub-diploid DNA content was found in about 55% in DMBC11 cells and 34% in DMBC12 cells after combined treatment with olaparib and DTIC for 48 hours, and this effect was further increased with the next dose and prolonged treatment. This might indicate that these compounds were more likely to induce cell death than cytostatic effects in melanoma cells, which was further confirmed by cell cycle analysis. Cell cycle arrest was not clearly visible in olaparib or DTIC treated melanoma cells, and only a modest fraction of cells treated with olaparib + DTIC accumulated in G2/M (Figure 3C).

Soft agar was used as a semisolid support to obtain spatially distinct colonies. When used alone, DTIC and olaparib reduced the number of colonies (Figure 3D). When drugs were used in combination, the clonogenic efficiency was further reduced.

**Figure 4:**
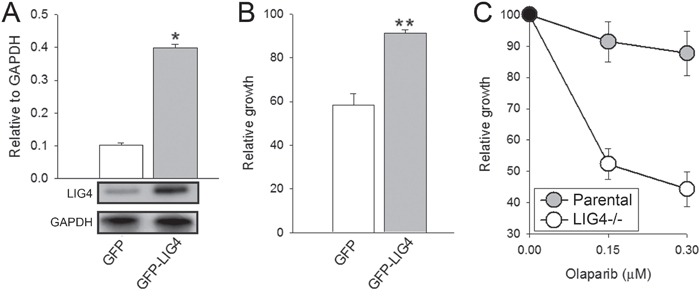
Sensitivity to olaparib depended on LIG4 expression levels **A.** Quantification of normalized LIG4 levels to GAPDH in total cell lysates obtained from GFP+ DBM11 cells transfected with expression plasmids encoding GFP or GFP and LIG4. Bars represent mean percentage volume intensity ± SD from 3 experiments; *p < 0.001 in comparison with GFP. Representative Western blots of the expression of LIG4 and GAPDH (loading control) are shown. **B.** The effect of olaparib on viability of DMBC11 cells transfected with GFP or GFP + LIG4. Results represent mean ± SD from 3 independent experiments; **p < 0.05 in comparison with GFP. **C.** The effect of olaparib on viability of Nalm6 parental and Nalm6 *LIG4−/−* pre-B cells. Results represent mean ± SD from 3 independent experiments.

To validate the importance of reduced level of LIG4 on the susceptibility of melanoma cells to olaparib, LIG4 was ectopically expressed in DMBC11 cell line (Figure 4A). Elevated expression of LIG4 reduced the sensitivity of DMB11 cells to olaparib (Figure 4B). Moreover, *LIG4−/−* pre-B cells were more sensitive to olaparib treatment than parental cells expressing endogenous LIG4 (Figure 4C).

### Olaparib and DTIC, used alone or in combination, increase the number of DSBs in patient-derived LIG4 deficient melanoma cells

**Figure 5:**
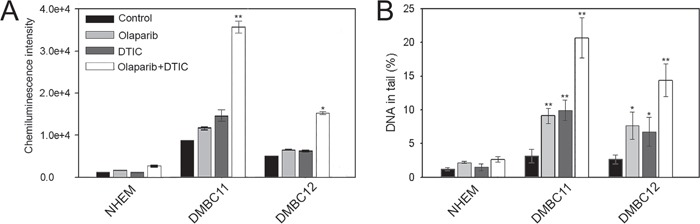
Olaparib and/or DTIC induced DSBs in melanoma cell lines (DMBC11, DMBC12) Cells were treated with 5 μM olaparib and/or 2 mM DTIC for 48 hrs (comet assay) and 120 hrs (γ-H2AX). **A.** The mean values ± SD of γ-H2AX were calculated from 3 ELISA experiments performed in triplicates. **B.** The mean percentage ± SD of DNA in the tails of comets in neutral conditions acquired from one hundred cells/group from 3 experiments. *p<0.05 and **p<0.001 in comparison with control.

In normal melanocytes the level of phosphorylated γ-H2AX, which marks DSBs [12], remained unchanged after the treatment. However, DMBC11 and DMBC12 cell lines showed increased levels (5- or 2-fold, respectively) of phosphorylated γ-H2AX in comparison to melanocytes (Figure 5A). Moreover, combined treatment approximately doubled the level of phosphorylated γ-H2AX in both melanoma cell lines in comparison to cells treated with either drug alone.

The neutral comet assay was also used to measure the ability of olaparib and/or DTIC to induce DSBs as described before [13]. DMBC11 and DMBC12 cell lines treated with individual drugs showed increased intensity of DNA tail in comparison to melanocytes indicating accumulation of DSBs (Figure 5B). Moreover, combination of olaparib and DTIC caused more DSBs that individual drug.

### Olaparib and DTIC combination reduces melanoma growth in NSG mice

**Figure 6:**
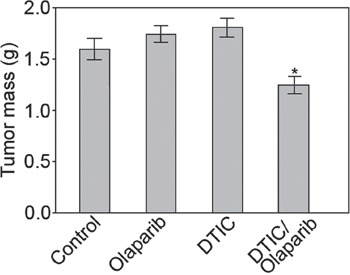
Combination of olaparib and DTIC reduced the growth of human melanoma in immunodeficient mice NAG mice were injected s.c. with DMBC11 melanoma cells followed by the treatment with olaparib (35 mg/kg twice a day), DTIC (8 mg/kg every second day), or olaparib + DTIC. Data represent mean ± SD of tumor mass from 2 independent experiments, *p<0.05 in comparison with untreated mice.

Sub-optimal doses of olaparib or DTIC did not reduce the growth of DMBC11 cells in NSG mice (Figure 6). Interestingly, the combination of olaparib and DTIC exerted modest, but statistically significant anti-melanoma effect. Stronger effect would probably require optimization of the treatment protocol.

## DISCUSSION

Synthetic lethality is a phenomenon occurring when simultaneous depletion of a pair of genes or gene products is required for cell death to occur. For example, cells harboring BRCA1/2 inactivating mutations are sensitive to PARP1 inhibitors [14, 15]. Therefore, PARP1 inhibitors may be highly effective drugs in variety of tumors with germline or somatic defects in DNA damage repair genes. In the present study we showed that PARP1 inhibitor olaparib applied alone and in combination with DTIC (a drug used in melanoma treatment) was effective against melanoma cells displaying downregulation of LIG4 without affecting normal melanocytes. This effect was associated with accumulation of toxic DSBs, implicating olaparib-mediated synthetic lethal effect in LIG4 deficient melanoma cells. Downregulated LIG4 and/or Artemis were detected before in cell lines established from high-risk neuroblastomas and therapy-resistant breast carcinomas [16, 17]. However, these studies did not establish that sensitivity to PARP1 inhibitors depended on inhibition of LIG4. Our work for the first time demonstrates that downregulation of LIG4 in melanoma cells is directly responsible for enhanced sensitivity to olaparib.

Our results suggest the new therapeutic approach against melanomas based on synthetic lethality which exploits the reduced levels of LIG4, an essential component of D-NHEJ that performs the final ‘end processing' step of DSB repair [18]. When LIG4 expression is reduced, D-NHEJ repair is performed inefficiently, and additional inhibition of PARP1-dependent B-NHEJ, BER and/or replication fork restart by olaparib could result in accumulation of toxic DSBs [5, 7–9]. Altogether, we postulate that D-NHEJ deficiency caused by downregulation of LIG4 could be synthetically lethal with B-NHEJ deficiency induced by PARP1 inhibitor. This hypothesis is supported by the results showing that PARP inhibitors were selectively toxic to LIG4-deficient melanoma and leukemia cells (this work) and that they increased DNA damage induced by radiation exposure in *LIG4−/−* HCT116 colon carcinoma cell line [19].

**Figure 7:**
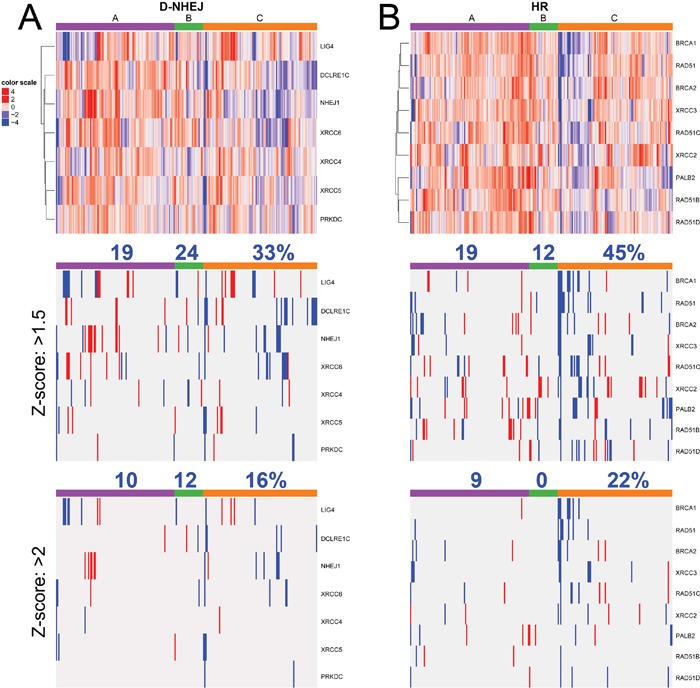
Transcriptome microarrays analysis of expression of the genes in A. D-NHEJ pathway, and B. HR pathway from 229 melanoma cell lines established from patients manifesting the following phenotypes: A- proliferative, C- invasive, and B- intermediate Percent above column color bar represents number of samples with at least one downregulated gene within the phenotype group.

Although downregulation/mutation of LIG4 (and its partner XRCC4) was detected only in approximately 7% of cutaneous melanomas in TCGA database (Figure 1), inhibition/inactivating mutation of other members of D-NHEJ potentially impairing DSB repair activity by the pathway were detected, too [20]. Moreover, transcriptome analysis by microarrays of 229 melanoma cell lines detected downregulation of at least one member of D-NHEJ pathway (including LIG4) in numerous samples established from patients manifesting different stages of malignancy (Figure 7A). The 229 melanoma cells were grouped by their molecular phenotype, proliferative, intermediate and invasive. The proliferative phenotype is defined by high expression of MITF and low expression of WNT5A, the invasive phenotype is defined by low expression of MITF and high expression of WNT5A, and intermediate phenotype have approximately equal expression of MITF and WNT5A. From the analysis, it seems that the invasive phenotype has greater downregulation in the D-NHEJ genes than proliferative, therefore selected melanomas with an invasive phenotype should display enhanced sensitivity to PARP1 inhibitors.

In addition, multiple melanoma samples displayed downregulation of at least one gene in HR pathway (Figure 7B) with higher frequency in the invasive phenotype suggesting their sensitivity to synthetic lethality triggered by PARP1 inhibitors [21]. In concordance, inhibition of histone deacetylases class I resulted in suppression of HR due to down-regulation of RAD51 and FANCD2 and sensitized malignant melanoma cells to a synthetic lethal effect of olaparib combined with alkylating drug temozolomide [22].

Despite downregulation/mutations of DSB repair genes detected in numerous samples in TCGA and transcriptome microarray databases, melanomas typically do not respond well to DNA damaging agents. Perhaps the degree of downregulation of DNA repair genes is not strong enough to increase the sensitivity to chemotherapeutics in clinical settings. However, as suggested by this work, the effect may become clinically relevant in repair-deficient cells when a genotoxic drug is combined with PARP1 inhibitor, which further enhances DNA damage beyond a reparable threshold.

In summary, PARP1 inhibitor seems to offer additional treatment opportunity to pre-selected melanomas displaying LIG4 (and/or XRCC4) deficiency. In addition, analyses of the already existing databases strongly suggest that numerous melanomas could be sensitive to personalized medicine-guided PARP1 inhibitor-mediated synthetic lethality due to their putative deficiencies in DNA repair pathways. This speculation is supported by phase II study showing almost doubled (although not statistically significant) progression-free survival of the patients with metastatic melanoma treated with veliparib + temozolomide compared with placebo + temozolomide. Perhaps personalized medicine approach is necessary to pre-select patients with melanomas predisposed to synthetic lethality mediated by PARP1 inhibitor.

## MATERIALS AND METHODS

### *In vitro* cell cultures

Melanoma cell lines derived from surgical specimens of nodular (DMBC2, DMBC8, DMBC9, DMBC10, DMBC12) and superficial spreading melanoma (DMBC11) were established in the Department of Molecular Biology of Cancer. The study was approved by the Ethical Commission of the Medical University of Lodz, and informed consent was obtained from all patients. Melanoma cells were cultured in Stem Cell Medium (SCM) as described elsewhere [23, 24]. Normal Human Melanocytes (NHEMs – Ad, Lonza) were cultured in Melanocyte Cell Basal Medium (MBM) (CC-3250, Lonza) supplemented with growth supplements according to the manufacturer's protocol. Nalm6 parental and Nalm6 LIG4−/− pre-B cells were purchased from HORIZON (www.horizondiscovery.com) and cultured in RPMI medium with 10% FBS (Lonza) and antibiotics (100 IU/ml penicillin, 100 mg/ml streptomycin (Gibco) at 37°C in a humidified atmosphere containing 5% CO_2_.

### Drug treatment

Melanoma cells and NHEMs were plated at a density of 1 × 10^5^ viable cells per well in a 6-well plates one day before drug treatment. Cells were cultured with 5 μM olaparib (Selleckchem), 2 mM dacarbazine (DTIC) (Sigma Aldrich), olaparib + DTIC, or vehicle. After 48 hours, half the cell suspension from each well was taken to determine cell viability after propidium iodide (PI) staining and cell cycle analysis. Following this, 1 ml of fresh medium containing drugs at appropriate concentrations was added to the remaining cell culture for additional 72 hours of culturing.

### Clonogenic assay

Melanoma cells were first incubated with compounds at indicated concentrations for 48 hours and then for 72 hours. Then, 1000 single viable cells were transferred to soft agar and clonogenic assay was performed as previously described [23].

### Flow cytometry

Flow cytometry and propidium iodide (PI) staining was used to assess changes in viability and cell distribution in cell cycle phases. Cells were analyzed using a FACSVerse flow cytometer (Becton Dickinson, San Jose, California, USA). ModFit LT 3.3 software (Verity Software, Topsham, Minnesota, USA) was used to calculate the percentage of cells in each cell-cycle phase and FACSuit software (Becton Dickinson) was used to calculate the percentages of dead cells in subG1.

### Ectopic expression of LIG4

Melanoma DMBC11 cells were transfected with plasmid pCMV6-AC-GFP with cloned human LIG4 cDNA (OriGene Technologies) using lipofectamine 3000 (Invitrogen) according to the manufacturer's protocol. GFP+ cells were sorted after 48 hrs and used for the experiments.

### Transcriptome microarrays analysis

Microarray data was obtained from NCBI GEO and analyzed for phenotype classes proliferative, intermediate and invasive as described in Widmer et al [25]. Microarray was subset for D-NHEJ genes and HR genes. Z-score cutoffs were set at 1.5 and 2 to detect upregulated and downregulated genes. Samples with at least one downregulated gene were counted.

### RNA isolation, cDNA synthesis and Real-Time PCR

Isolation and purification of RNA was performed using total RNA isolation kit (A&A Biotechnology). Subsequently, RNA was transcribed into cDNA using SuperScript II Reverse Transcriptase (Invitrogen Life Technologies, Carlsbad, California, USA). qRT-PCR was performed using TaqMan^®^ Real-Time PCR Master Mix (Life Technologies) and Agilent Technologies Stratagene Mx300SP working on MxPro software. TaqMan probes (Life Technologies) were used to analyze 8 genes whose products are essential for DSB repair pathways (*BRCA1*, *LIG3*, *LIG4*, *PALB2*, *PARP1*, *PRKDC*, *RAD51*, *XRCC6*), and *18S RNA* (Life Technologies) was included as the reference gene. The cycling parameters were 95°C for 10 minutes, 30 cycles of 95°C for 15 seconds and 60°C for 60 seconds.

### Western blot analysis

Cell lysates were obtained by incubating a cell pellet with RIPA buffer for 30 minutes. Lysates were than resolved by SDS-PAGE. The proteins were transferred onto an Immobilon-P PVDF membrane (Millipore), which were blotted overnight with primary antibodies recognizing GAPDH, DNA LIG4 (Santa Cruz Biotechnologies), Ku70, RAD51 or PARP1 (ThermoFisher Scientific). This was followed by 1 h incubation with secondary antibodies conjugated with HRP (Anti-Mouse and Anti-Rabbit antibodies, Cell Signaling).

### ELISA measurement of γ-H2AX

Cell lines DMBC11, DMBC12 and NHEMs were cultured with vehicle or with drugs on black 96-well plates with a clear bottom. Analysis of the level of phosphorylated histone γ-H2AX was performed using an H2AX Phosphorylation Assay Kit (Millipore, Billerica, MA, USA according to the protocol. Chemiluminescence detection was performed using attached HRP-substrates using a GloMax-Multi device (Promega). Bleomycin at 35 μM for 30 min was used as a control.

### Neutral comet assay measurement of DSBs

Cells were cultured with vehicle or drugs for 48 hours and analyzed by neutral version of comet assay to detect DSBs as described before with modifications [13]. Briefly, cells were suspended in 0.75% LMP agarose and casted onto microscope slides precoated with 0.5% NMP agarose. The cells were then lysed for 1 h at 4°C in a buffer consisting of 2.5 mM NaOH, 100 mM EDTA, 1% Triton X-100, 10 mM Tris, pH 10. After the lysis the slides were placed in an electrophoresis unit, DNA was allowed to unwind for 20 min in the electrophoresis buffer consisting of 100 mM Tris and 300 mM sodium acetate at a pH adjusted to 9.0 by glacial acetic acid. Electrophoresis was conducted in this electrophoresis buffer at 4°C for 60 min at an electric field strength of 0.41 V/cm (100 mA). The slides were then washed in water, drained and stained with 2 μg/ml of DAPI and examined at 200× magnification in an Eclipse fluorescence microscope (Nikon, Tokyo, Japan) attached to COHU 4910 video camera (Cohu, San Diego, CA, USA) equipped with a UV-1 filter block consisting an excitation filter (359 nm) and a barrier filter (461 nm) and connected to a personal computer-based image analysis system, Lucia-Comet v. 5.41 (Laboratory Imaging, Praha, Czech Republic). Fifty images were randomly selected from each sample and the percentage of DNA in the tail of comets (% tail DNA) was measured. The mean value of the % tail DNA in a particular sample was taken as an index of DSBs in the sample.

### Xenograft experiments

24 NSG mice were injected subcutaneously under the right scapula with 1×10^5^ melanoma cells previously suspended in Matrigel. After 4 days tumor-bearing mice were randomly assigned into four groups; untreated, and treated intraperitoneally either with olaparib (35 mg/kg bodyweight twice a day, diluted in DMSO), DTIC (8 mg/kg bodyweight every second day, diluted in PBS) or olaparib with DTIC (same dosing as in monotherapy) for 24 days. After the end of experiment tumors were collected and weighted. The study was approved by the local Ethical Committee.
